# Efficient phenotyping of banana (*Musa* spp.) germplasm for resistance to five biotic constraints using Partially replicated (P-rep) design

**DOI:** 10.1371/journal.pone.0361182

**Published:** 2026-10-23

**Authors:** Reagan Kanaabi, Gerald Mwanje, Charles Serebe, Happyness Mpanda, Kennedy Jomanga, Brigitte Uwimana, Marnik Vuylsteke, Allan Brown, Rony Swennen, Gloria Valentine Nakato

**Affiliations:** 1 International Institute of Tropical Agriculture (IITA), Namulonge-Sendusu, Uganda; 2 International Institute of Tropical Agriculture (IITA), c/o Nelson Mandela African Institution of Science and Technology (NM-AIST), Arusha, Tanzania; 3 Gnomixx B.V., Merelbeke-Melle, Belgium; 4 KU Leuven, Department of Biosystems, Willem De Croylaan 42, Heverlee, Belgium; National Cheng Kung University, TAIWAN

## Abstract

Banana breeding programs prioritize improving yield with resistance to biotic stresses while maintaining fruit quality. This study focused on identifying genotypes with resistance to five major biotic constraints including *Cosmopolites sordidus*, *Radopholus similis*, banana *Xanthomonas* wilt, *Fusarium oxysporum* f. sp. *cubense* – Race 1 and *Pseudocercospora fijiensis*. A diverse set of diploid and tetraploid genotypes was phenotyped under controlled screenhouse and field conditions to identify potential sources of. A partially replicated (P-rep) design, was used to accommodate unequal plant availability arising from asynchronous multiplication. P-rep design enabled robust phenotypic comparisons and identification of genotypes expressing resistance to multiple biotic stresses. Several diploid genotypes exhibited resistance to multiple constraints, highlighting their potential as parental lines for resistance introgression and broadening the genetic base for banana improvement. Leveraging on the efficiency and precision of the P-rep experimental design, these findings accelerate the phenotyping of genotypes.

## Introduction

Banana is an important staple and cash crop in East and Central Africa (ECA), with major production constraints imposed by banana weevil (*Cosmopolites sordidus*) [1–2]), root burrowing nematode (*Radopholus similis*) [3], Banana *Xanthomonas* wilt (BXW) [4,5], Fusarium wilt [6,7] and Sigatoka leaf diseases [8,9]. While chemical and cultural control methods are available for some of these constraints, the most effective and economically affordable solution to small scale farmers is the use of resistant varieties [10–13].

Resistance has been reported in wild and partially improved banana cultivars, but results are often difficult to compare because studies vary in controls, phenotyping methods, inoculum levels, biotypes, and how well experimental error is managed. This inconsistency makes parental selection uncertain, especially when terms like partial resistance are used loosely. Because crossbreeding is a long-term venture, precise phenotyping of parents is essential, yet genetic information remains limited and QTL findings are not routinely applied in most breeding programs [14,15].

Through crossbreeding, genetic gains are achieved by intercrossing elite parental lines with desirable attributes and, where necessary, by introgressing resistance alleles from unadapted germplasm into elite backgrounds to create favorable trait combinations in the next generation [16]. Conventional breeding seeks to capture genetic variation while accounting for environmental effects through appropriate experimental design and analysis [17]. In practice, phenotyping is often the main bottleneck, yet it strongly influences the precision of statistical models and the reliability of breeding decisions [18]. This is why controlled environments are commonly used for phenotyping, as they may limit uncontrolled environmental variation and improve the estimation of genetic effects [19]. However, controlled environments may fail to reproduce key field drivers of trait expression [20,21] and may not adequately represent G × E (genotype x environment) effects [22]. The choice between field and controlled phenotyping therefore depends on the target trait, its heritability, and the purpose and logistics of data collection [23].

In banana, experimental designs are important because obtaining sufficient, uniform planting material for large phenotyping trials remains a major challenge. Even under tissue-culture conditions, plant availability is often genotype dependent, as multiplication rate, rooting ability, contamination losses, acclimatization success and growth rate vary considerably among genotypes. As a result, large experimental sets cannot be established with balanced numbers of plants at the required time. Incomplete block designs help address this challenge by reducing environmental heterogeneity within smaller blocks and improving precision at lower cost [24]. Where replication is limited by scarce or uneven plant supply, partially replicated (P-rep) designs provide a practical compromise by fully replicating a subset of genotypes while partially replicating, or not replicating, other genotypes [25,26]. The appropriate experimental design ensures that observed differences among genotypes reflect true genetic potential and not environmental noise, improves accuracy and precision, enables appropriate modelling of phenotypic data, strengthens the assessment of G x E interactions, promotes efficient use of limited resources, and ultimately supports reliable selection decisions.

We assembled 69 widely used parental genotypes from the Matooke, Mchare, and Plantain breeding programs of IITA (International Institute of Tropical Agriculture) in Uganda, Tanzania, and Nigeria, and evaluated them using P-rep to identify parents with useful resistance traits for breeding. However, the number of parental genotypes phenotyped varied among biotic constraints.Top of Form

## Materials and methods

### Establishment of experimental plants

Phenotyping was conducted at IITA-Arusha, Tanzania (3° 24’ 7” S; 36° 47’ 51” E, 1208 m above sea level) and IITA-Sendusu, Uganda (0°31′30″ N; 32°36′54″ E, 1260 m above sea level). Although two locations were used across the study, each biotic constraint was evaluated at a single location and not a multi-location evaluation. *FOC*-R1 was evaluated at IITA-Arusha in Tanzania, while *C. sordidus*, *R. similis, P*. *fijiensis* and *Xvm* were evaluated at IITA-Sendusu in Uganda*.* For each constraint, phenotyping was conducted over multiple years between 2021 and 2025.

Parental genotypes from Matooke, Mchare and Plantain breeding programs were evaluated for resistance to weevils (*Cosmopolites sordidus*) and nematodes *Radopholus similis* infestation as well as infection by BXW (caused by *Xvm*), banana fusarium wilt (caused by *Fusarium oxysporum* f. sp. *cubense* (*FOC*-R1) and black Sigatoka (caused by *Pseudocospora fijiensis*). Genomic composition and ploidy of each genotype are shown in S1 Table. Landrace cultivars were used as susceptible controls, namely, Mbwazirume (Matooke AAA), Mchare (Mchare, AA), and Obino Lewai (Plantain, AAB). The wild banana, Calcutta 4 (AA), was used as a resistant check for weevils, *FOC*-R1, and black Sigatoka while SH 3142 (AA) and Monyet (AAAA) were used as resistant checks for nematodes and *Xvm*, respectively.

All experimental plants were generated in tissue culture laboratories in Arusha, Tanzania and Sendusu, Uganda. Healthy peeper and sword suckers were selected from vigorous, disease free mother plants of each genotype in the field. A peeper is a small sucker that has just emerged from the soil while the sword sucker is more developed than a peeper and has narrow, sword like leaves. The suckers were washed thoroughly, the outer leaf sheaths were removed, and the corm tissue was trimmed to obtain a 6–8 cm explant containing the shoot apex. Explants were surface sterilized in 15% (v/v) Jik (3.5% sodium hypochlorite) household bleach supplemented with one drop per 50 mL Tween 80 for 15 min, followed by immersion in 70% ethanol for five min, and then in 10% (v/v) Jik household bleach containing Tween 80 for 25 min. Thereafter, the explants were rinsed three times with sterile distilled water. At initiation, the shoot tip meristem, together with three to four surrounding leaf sheaths, was placed upright on Murashige and Skoog propagation medium [27] and incubated in a growth room under light. To assess the occurrence of bacterial contamination, a small piece of tissue from each explant was aseptically placed on bacterial growth medium and incubated under white fluorescent light conditions. The plates were examined after two to three days for fast growing bacterial contaminants and maintained for up to 8 weeks to detect slow growing bacteria. Explants that remained free of contamination were transferred to fresh propagation medium after two to three weeks. Thereafter, subculturing was carried out at two to three week intervals to remove necrotic tissue and divide explants to stimulate shoot proliferation. Further subdivision into smaller explants was performed every three to four weeks until shoots had developed pseudostems and at least three leaves. For rooting, pseudostems were trimmed to 1–2 cm and transferred to rooting medium [28] for 3–4 weeks, producing plantlets 2–5 cm in height with three to five leaves and well-developed roots suitable for acclimatization. All plantlets were hardened off in a humid chamber and nursery for three months before experimental establishment.

*C. sordidus*, *R. similis*, *Xvm* and *FOC*-R1 were evaluated in pot trials in the screenhouse via artificial inoculation. Plants were maintained in a screenhouse at 25°C with regular watering. *Pseudocercospora fijiensis* was evaluated in the field in a *P*. *fijiensis* hotspot under natural infection. A cooking EAHB matooke, Enzirabahima, was used as a spreader row to ensure enough inoculum in the field.

### Experimental design

We adopted the P-rep design nested into multiple experiments called batches evaluated at different times for *C*. *sordidus*, *R*. *similis*, *Xanthomonas vasicola* pv. *musacearum* **(***Xvm*) and *FOC*-R1 [25,29]. There were five batches for *C*. *sordidus* and *Xvm*, four batches for *R*. *similis*, three cycles for *P*. *fijiensis* and two batches for *FOC*-R1. On average, there were 20 genotypes per batch. Genotypes in each batch were not necessarily similar for each biotic constraint. There were at most four plants per genotype per block with each test genotype appearing in one or two of the blocks while the checks and controls appeared in all three blocks. For *P*. *fijiensis*, data were collected by cycle. A cycle is the period from planting to harvesting of the initial banana plant or from sprouting from the mother corm to harvesting for subsequent ratoons in a banana crop. On average, each genotype had eight pseudo replications for each constraint. Response variables for the targeted biotic constraints are indicated in Table 1. The number of parental genotypes phenotyped for each biotic constraint are indicated in Table 1.

**Table 1:** Biotic constraints, their corresponding response variables and total number of genotypes and plants phenotyped for each.

Biotic constraint	Response variable	No. of Parental genotypes	No. of test plants
*C*. *sordidus*	Percentage total cross-sectional damage of corm (%TXD)	65	899
*R*. *similis*	Percentage root necrosis (%TRN)	59	721
*Xvm*	Area under disease progressive curve (AUDPC)	64	267
*FOC*-R1	Internal corm symptoms (ICS)	48	734
*P*. *fijiensis*	Area under disease progressive curve (AUDPC)	60	734

**Note:** Number of Parental genotypes are those that were evaluated for each biotic constraint, excluding the checks. Resistant and susceptible genotypes were included as checks in the respective phenotyping assays.

### Genomic relationship between parental genotypes

The phenotyped 69 parental genotypes and an additional seven not assessed in this study, were genotyped as part of the study on diversity in *Musa* breeding germplasm, comprising 856 accessions from the International Institute of Tropical Agriculture (IITA), the National Agricultural Research Organization of Uganda, the Brazilian Agricultural Research Corporation (Embrapa), the National Research Centre for Banana of India (NRCBI), and additional material from the banana International Transit Center [30,31]. Genotyping was carried out using the DArTSeq platform as described by Akech et al. [30]. Briefly, leaf samples were shared with DArT Diversity Arrays Technology (DArT®) Australia through Nature Source Improvement Plants (NSIP). Genome complexity reduction was carried out using *PstI* and *Mse*I restriction enzymes. Sequencing was performed on the Illumina HiSeq 2000 platform, at 1.25 million reads. Single nucleotide polymorphism (SNP) markers were identified and their physical positions determined by aligning the sequences to the 4^th^ version of the double haploid Pahang banana reference genome [32]. The genotypic data were filtered to a maximum of 20% missing marker data, minimum of 95% call rate, and 5% minor allele frequency. The resulting genotypic data set comprised 13306 SNP markers across the genotyped parental materials.

### Data collection

#### *C*. *sordidus* damage assessment.

Weevil damage was assessed according to Mwanje et al. [33]. At three months post hardening, plants were infested with three female and three male weevils at the base of each plant in a bucket, and buckets sealed off with weevil-proof nets to prevent escape [33,34]. At 60 days post inoculation, plants were uprooted, and corm damage assessed using the cross-section method [35]. Corm damage was scored for each cross-section as percentage of the damaged area of the cortex (upper and lower outer cross-sectional damage) and the inner cylinder (upper and lower inner cross-section damage) [36]. The percentage total cross-sectional damage (TXD) was estimated by taking the average of the four readings of the cortex and inner cylinder [37].

#### *R. similis* damage assessment.

Nematode damage was assessed according to Kisitu et al. [38]. Each experimental plant was inoculated with 1000 nematodes [39]. Plants were not watered for 48 hours post inoculation to minimize displacement of the introduced nematodes and encourage penetration into the roots. The experiment was terminated at eight weeks post-inoculation and data on root necrosis and nematode population density recorded [38]. Root necrosis was assessed by selecting five functional roots per plant, cutting them into 10 cm segments, splitting them longitudinally, and visually estimating the percentage of necrotic cortex. The scores from the five root pieces were summed to obtain a percentage root necrosis index (RNI) calculated as the percentage of area with necrotic root cortex on five randomly selected live roots [40]. Nematodes were extracted following the Baermann funnel technique for nematode extraction [41]. All roots from an individual plant were chopped into 0.5 cm pieces and homogenized. A 10g sample of the homogenate was macerated used in nematode extraction. The extracted nematodes were counted three times using 2 mL aliquots from a 25 mL suspension and densities expressed per 100 g fresh root mass. Population densities consisted of juveniles, females, and males.

### BXW severity assessment

*Xanthomonas vasicola* pv. *musacearum* (*Xvm*) inoculum was prepared by culturing *Xvm* strain U40L2 [5] in YPG broth at 28°C for 48 hours and adjusting the inoculum to 108 CFU/mL (approximately 0.5 OD600) using a 2009 Thermo Scientific Evolution 60S UV-Visible spectrophotometer. Three months post-planting, experimental plants were inoculated with 1000 μL of the *Xvm* inoculum at the youngest leaf according to Ssekiwoko et al. [42]. Plants were monitored weekly for four months, and symptoms typical of BXW were scored [43]. Disease severity index (DSI) was scored on a 0–3 scale, based on Winstead and Kelman [44] and modified by Nakato et al. [5]: 0 = no symptoms, 1 = necrosis of inoculated leaf, 2 = wilting of uninoculated leaves, and 3 = death of the entire plant. The area under the disease progress curve (AUDPC) was computed using the disease severity index as highlighted in Nakato et al. [5].

### *FOC*-R1 severity assessment

A known *FOC*-R1, VCG-0125, cultured on Potato Dextrose Agar (PDA) until full mycelial growth was used to inoculate sterilized millet kernels, which were incubated for 14 days at 25°C. Plants were grown in pots containing a soil mixture, with 25 g of infected millet kernels mixed into the lower third and with the roots in direct contact. The remaining two-thirds of the pot were filled with sterile forest soil. Disease evaluations included external symptoms such as Leaf Symptom Index (LSI), and Pseudostem Splitting (PSS) recorded biweekly for three months, and internal symptoms such as Rhizome Discoloration Index (RDI), assessed at the end of three months. LSI was rated on a 1–5 scale and PSS on a 1–3 scale adapted from Piepho and Williams [26]. RDI was scored on a 1–6 scale based on vascular discoloration. Disease Severity Index (DSI) was calculated following Dita et al. [45] where 0 = no vascular discoloration in the corm, 5 = total discoloration of the vascular tissue in the corm and intermediate scores representing increasing discoloration of the vascular tissues within the corm. Genotypes were classified as resistant or susceptible based on LSI and RDI ratios.

#### *P*. *fijiensis* severity assessment.

The experimental field was established in a location with high disease pressure for natural infection. We also planted spreader rows of a highly susceptible genotype around the experiment to further concentrate the inoculum. Agronomic practices similar to farmer practices were adopted, and leaf spots were not controlled. Data were collected for two successive cycles on the mother plant and first sucker.

Data collection began at six months post planting. Disease was scored at the vegetative, flowering, and harvesting stages by counting the number of standing leaves (NSL) and functional leaves. NSL refers to the total leaves on the plant at the time of assessment, whereas functional leaves are leaves with at least 50% photosynthetic area. Additionally, the severity of each leaf was visually rated according to Fouré [46] using a disease severity scale from 0–6, where 0 = no symptoms, 1=<1%; 2 = 1–5%; 3 = 6–15%; 4 = 16–33%; 5 = 34–50% and 6 = 51–100% of the leaf area covered [47]. The youngest leaf with visible streak symptoms (YLst) and the youngest leaf spotted (YLS) were recorded at the most advanced stage (SSD); the stage at which symptom progression stopped, YLst and YLS were recorded. The index of non-spotted leaves (INSL) was calculated using equation 1, and the disease severity index per plant was computed as indicated in Kimunye et al. [48].


$$\begin{array}{c} INSL=\frac{\text{YLS }-\;\text{1}}{\text{NSL}}\times \text{ 100} \end{array}$$ (1)

The AUDPC was computed using the disease severity index as highlighted in Kimunye et al. [48].

### Statistical analysis

Pest and disease trait data were analyzed using a conjugate Hierarchical Generalized Linear Model (HGLM) [49] using Genstat (24th Edition, VSN International), accounting for fixed genotype effects and random batch and block nested into batch **effects**. The **Poisson distribution** was assumed for the *C*. *sordidus*, *R*. *similis*, and *FOC*-R1 response variable, with a **log-link function; the distribution of the random effects was restricted to gamma distribution, with a log-link, to give a** HGLM model. Normal distribution was assumed for the *Xvm* and *P*. *fijiensis* response variable, with an identity link function; the additional random terms were constrained to follow a normal distribution and to have an identity link. In all cases, the dispersion parameter was set to be estimated, and residuals were checked for normality. Fixed genotype effects and their standard error were estimated as differences compared with the resistant control and expressed on the link scale. These genotype effects are visualized in forest plots generated using Python [50–51]. The significance of genotype effects was assessed by a t-test. Predicted values for a future observation and standard error based on the fitted HGLM, were expressed on the original scale.

### Hierarchical clustering using Ward’s Linkage method

Hierarchical clustering was performed using Ward’s linkage to group genotypes by multivariate similarity [52–54]. Variables were standardized prior to analysis, euclidean distances were computed, and clusters merged to minimize within-cluster variance. Results were visualized with a dendrogram, and the optimal number of clusters selected based on marked increases in linkage distance. All analyses were completed in Python using the SciPy and scikit-learn libraries.

### Principal component analysis

Principal component analysis (PCA) was performed using the ‘scikit-learn library’ in Python (version 3.8) [55]. Prior to analysis, the data were standardized using ‘StandardScaler’ to ensure each variable contributed equally to the principal components [55]. PCA was applied to the scaled data using PCA() function. The first few components explaining majority of variance were retained for visualization and interpretation.

### Genotype classification

Genotypes were classified according to their estimated response relative to the model derived estimates relative to the resistant control. Genotypes with point estimates on or to the left of the reference line were classified as resistant. Genotypes with estimates to the right of the reference line but with 95% confidence intervals overlapping the reference were classified as tolerant, whereas genotypes with estimates to the right of the reference line and 95% confidence intervals that did not overlap the reference were classified as susceptible.

## Results

### Genetic relationship between parental genotypes

The generated PCA summarized genetic variation among the parental genotypes, based on markers with the first two components explaining 31.8% and 10.1% of the genetic variance, respectively (Fig 1A). Hierarchical clustering grouped the banana genotypes into four distinct clusters that were consistent with the PCA outcome, pedigree and resistance patterns across the biotic constraints (Fig 1B). Cluster 1 comprised the plantain landrace Obino Lewai and the derived plantain hybrids, PITAs, and it was associated with resistance to *R*. *similis*, *Xvm* and *FOC*-R1. Cluster 2 was dominated by the diploids and wild types, mainly resistant to *R*. *similis* and *FOC*-R1. Cluster 3 genotypes showed resistance to *R. similis, FOC*-R1 and *P*. *fijiensis* while cluster 4 contained hybrids with multiple resistance traits, notably to *C*. *sordidus*, *R*. *similis* and *FOC*-R1.

**Fig 1:**
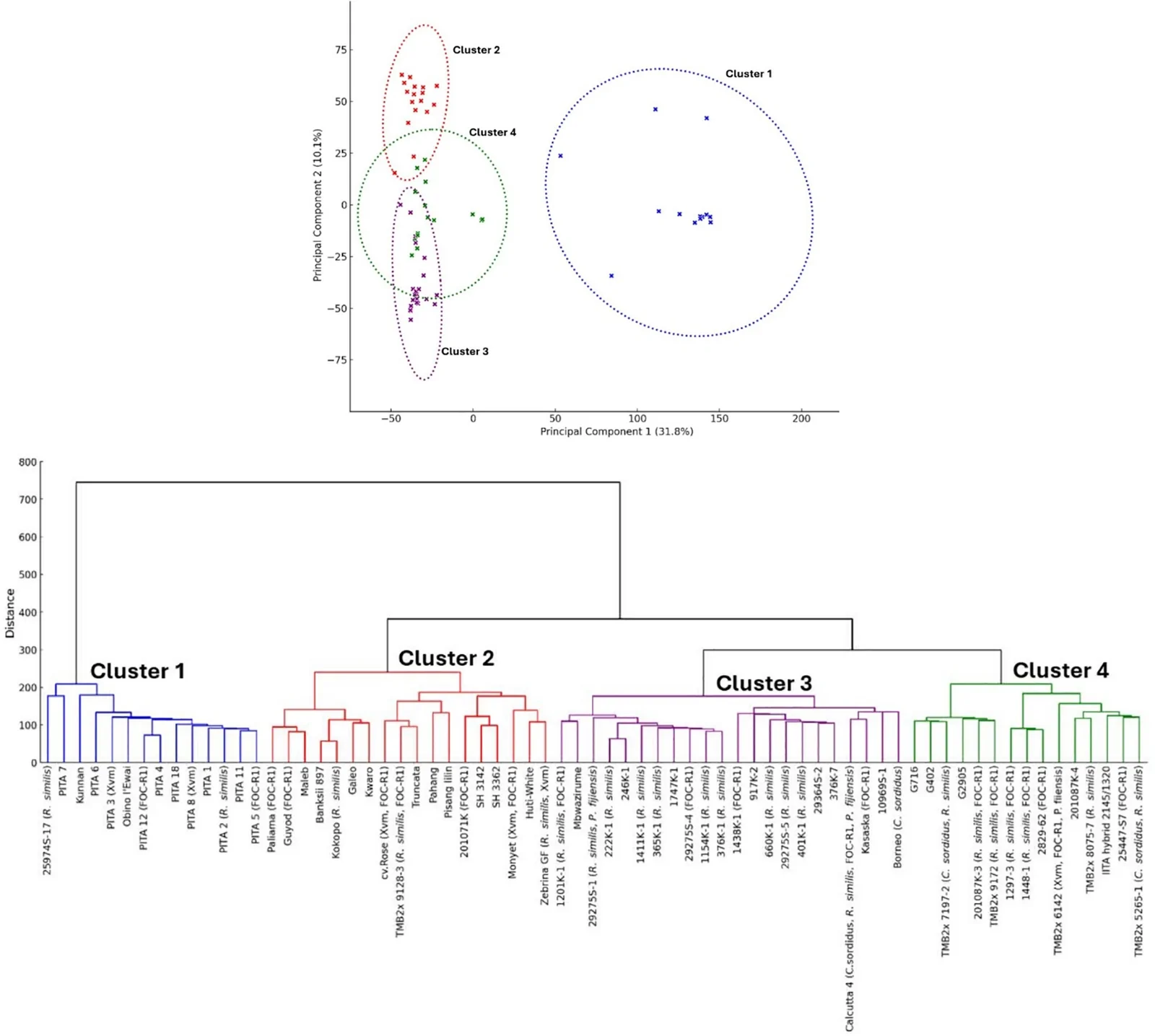
Cluster analysis of Genotypes using PCA and Hierarchical clustering. (A) The scatter plot visualizes the results of Principal Component Analysis (PCA). The first two principal components accounted for 41.9% of the total genetic variation. Each cross in the plot represents a genotype, colored according to their cluster assignment. (B) Dendrogram showing genetic relationships among the banana genotypes. Clustering was performed using Ward’s method and branches match the PCA-derived clusters. The vertical axis indicates the linkage distance, with lower heights reflecting higher genetic similarity.

### Responses to the biotic constraints

Fig 2 shows density distribution of genotype responses by batch for each constraint, with batches distinguished by color. Across constraints, there was an overlap in the density curves, although some batches diverged in location and spread, indicating batch-specific variability. A similar pattern for density curve overlap and batch-specific variability was observed for P. fijiensis across cycles (Fig 2E). In Fig 1B, the biotic constraint listed next to each genotype shows which one the genotype is resistant to.

**Fig 2:**
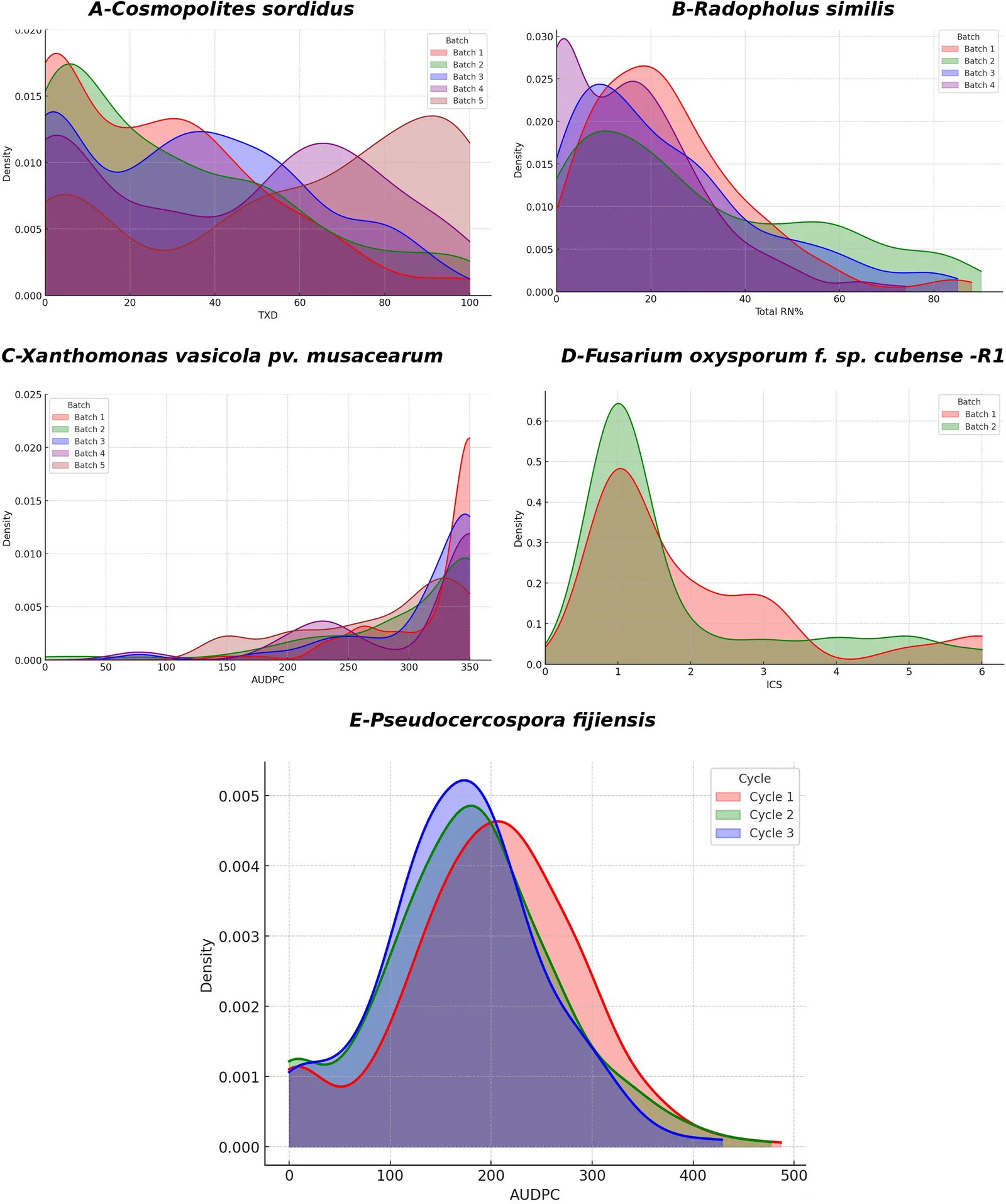
Density plots. (A) %Total cross-sectional damage for *C*. *sordidus*, (B) %Total root necrosis for *R*. *similis*, (C) AUDPC for *Xvm* by batches, (D) internal corm symptoms for *FOC*-R1 and (E) AUDPC for *P*. *fijiensis* by cycles.

Batch-specific control/check means provided biological reference points for interpreting genotype responses (S2 Table). Calcutta 4 consistently showed low levels of damage or disease for *C. sordidus*, *R. similis*, FOC-R1 and *P*. *fijiensis*, whereas the land races showed higher damage levels (S2 Table). Monyet showed lower AUDPC values for *Xvm* than the susceptible checks (S2 Table). These control means provide a biological reference for interpreting model-based genotype estimates on the original response scale.

### Genotype response to targeted pests and diseases

Conjugate HGLM models were fitted to estimate fixed genotype effects, reported as contrasts against the resistant control on the link scale with associated standard errors and visualized as forest plots (Fig 3 and 4). Based on the model derived estimates presented in the forest plots, parental genotypes were classified according to their response relative to the resistant control reference as explained under genotype classification. Landraces were used as references for biological interpretation but were excluded from the calculation of parental genotype frequencies and percentages. For *C. sordidus*, two of the 65 parental genotypes (3.1%) were resistant, while one (1.5%) was tolerant. For *R. similis*, 14 of the 59 parental genotypes (23.7%) were resistant, comprising nine diploids and six tetraploids, while 16 (27.1%) were tolerant (Fig 3). For *Xvm*, four of the 64 parental genotypes (6.3%) were resistant, while two (3.1%) were tolerant (Fig. 4). For *FOC*-R1, 12 of the 48 parental genotypes (25.0%) were resistant and 12 (25.0%) were tolerant. Resistance to *P. fijiensis* was not observed among the 59 parental genotypes under the forest-plot classification, although two genotypes (3.3%) were classified as tolerant (Fig. 4). The forest plots present model-based genotype estimates and 95% confidence intervals on the model link scale. For biological interpretation, batch-specific control means are provided in S2 Table, allowing the relative magnitude of genotype responses to be interpreted on the original biological scale.

**Fig 3:**
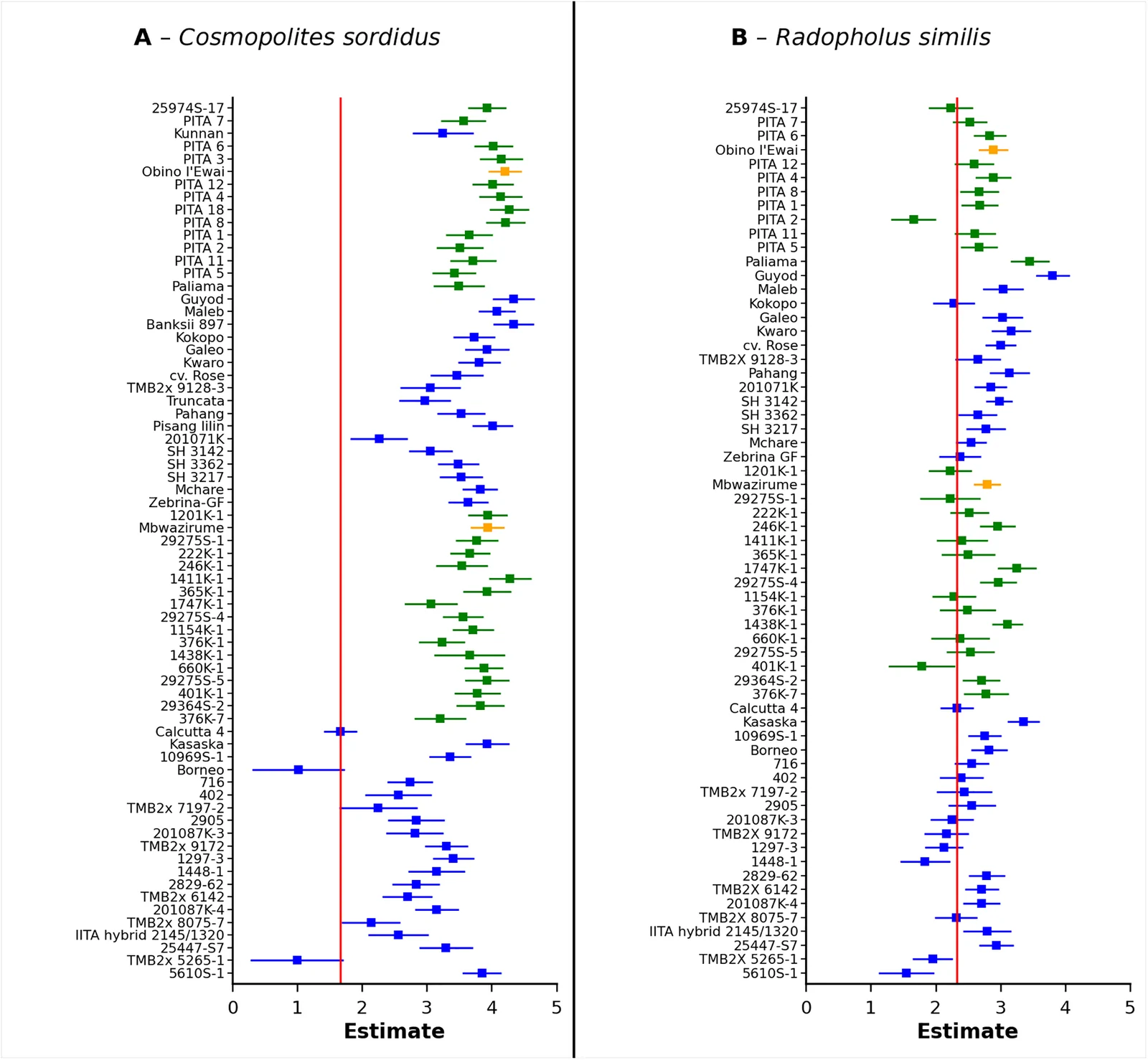
Forest plots. (A) % total cross-sectional damage (%TXD) for Cosmopolites sordidus (B) % root necrosis (%TRN) for Radopholus similis. Each square represents a genotype’s adjusted mean on the link scale, with horizontal lines indicating + /- the standard error. Diploid genotypes are in blue, triploids in orange and tetraploids in green. The red solid vertical line represents the reference value of Calcutta 4 as the resistant control. Genotypes positioned to the left of the reference line are more resistant than the reference genotype.

**Fig 4:**
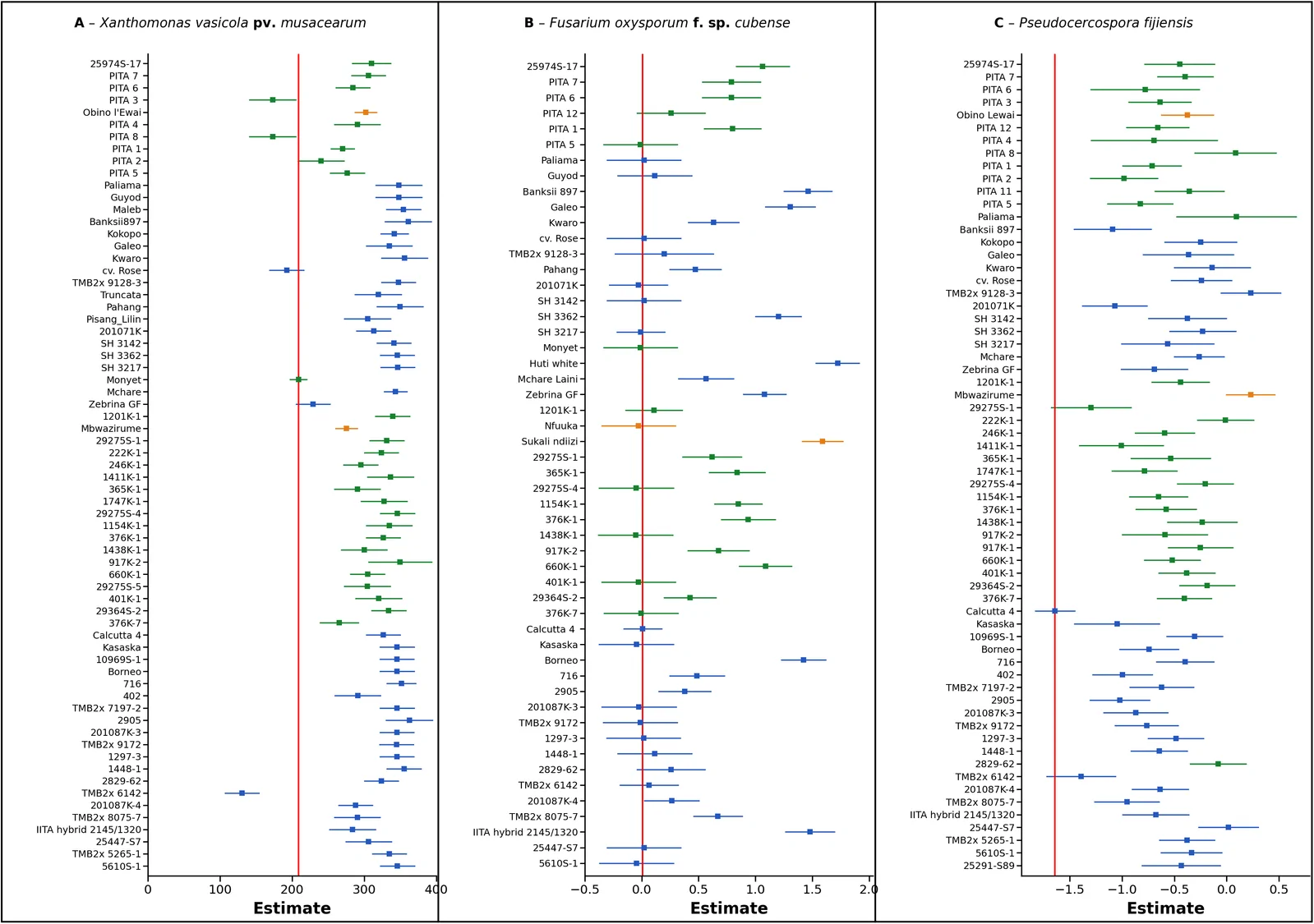
Forest plots. (A) AUDPC for Xanthomonas vasicola pv. musacearum. (B) internal corm symptoms (ICS) for FOC-R1. (C) AUDPC for Pseudocercospora fijiensis. Each square represents a genotype’s adjusted mean on the link scale, with horizontal lines indicating + /- the standard error. Diploid genotypes are in blue, triploids in orange and tetraploids in green. The red solid vertical line represents the reference value of Monyet as the resistant control for Xanthomonas vasicola pv. musacearum and Calcutta 4 as the resistant control for FOC-R1 and Pseudocercospora fijiensis. Genotypes positioned to the left of the reference line are more resistant than the reference genotype.

Forty-two genotypes were phenotyped for responses to all five biotic constraints. Of these, eight genotypes (201087K-4, Galeo, IITA hybrid 2145/1320, Kwaro, PITA 1, PITA 6, SH 3362, and 29364S-2) were susceptible to all five constraints, while the remaining 34 exhibited different combinations of resistance, tolerance, and susceptibility (Table 2). Several genotypes showed resistance or tolerance to more than one constraint. Calcutta 4 was resistant to *C. sordidus*, *R. similis*, FOC-R1, and *P. fijiensis*, but susceptible to *Xvm* (Table 2). TMB2x 6142 was resistant to *Xvm* and tolerant to FOC-R1 and *P. fijiensis*. 1297−3, 201087K-3, 5610S-1, 401K-1, and TMB2x 9172 were resistant to both *R. similis* and FOC-R1 (Table 2). 1448−1 and 1201K-1 were resistant to *R. similis* and tolerant to FOC-R1, while TMB2x 9128−3 was tolerant to both constraints. 201071K, 1438K-1, 29275S-4, 376K-7, and PITA 5 were resistant to FOC-R1, whereas 1154K-1 and 25974S-17 were resistant to *R. similis* (Table 2). TMB2x 8075−7 was tolerant to *C. sordidus* and resistant to *R. similis*, while Borneo was resistant to *C. sordidus*. cv. Rose was tolerant to FOC-R1 and resistant to *Xvm*, and Zebrina GF was tolerant to *R. similis* and *Xvm* (Table 2). 29275S-1 was resistant to R. similis and tolerant to P. fijiensis. Other genotypes showed single-constraint responses, including tolerance to R. similis in 716, 2905, 365K-1, 376K-1, 660K-1, and PITA 7; tolerance to FOC-R1 in 25447-S7, 2829−62, SH 3142, and Paliama; and resistance to FOC-R1 in Kasaska. No genotype was resistant or tolerant to all five biotic constraints (Table 2).

**Table 2:** Summary of resistance response of genotypes phenotyped for all five biotic constraints.

Genotypes	Ploidy	Cluster	Constraint				
			*C*. *sordidus*	*R*. *similis*	*Xvm*	*FOC*-R1	*P*. *fijiensis*
25974S-17	4	1	Susceptible	**Resistant**	Susceptible	Susceptible	Susceptible
PITA 7	4	1	Susceptible	**Tolerant**	Susceptible	Susceptible	Susceptible
PITA 6	4	1	Susceptible	Susceptible	Susceptible	Susceptible	Susceptible
PITA 1	4	1	Susceptible	Susceptible	Susceptible	Susceptible	Susceptible
PITA 5	4	1	Susceptible	Susceptible	Susceptible	**Resistant**	Susceptible
Paliama	4	2	Susceptible	Susceptible	Susceptible	**Tolerant**	Susceptible
Galeo	2	2	Susceptible	Susceptible	Susceptible	Susceptible	Susceptible
Kwaro	2	2	Susceptible	Susceptible	Susceptible	Susceptible	Susceptible
cv. Rose	2	2	Susceptible	Susceptible	**Resistant**	**Tolerant**	Susceptible
TMB2x 9128−3	2	2	Susceptible	**Tolerant**	Susceptible	**Tolerant**	Susceptible
201071K	2	2	Susceptible	Susceptible	Susceptible	**Resistant**	Susceptible
SH 3142	2	2	Susceptible	Susceptible	Susceptible	**Tolerant**	Susceptible
SH 3362	2	2	Susceptible	Susceptible	Susceptible	Susceptible	Susceptible
Zebrina GF	2	2	Susceptible	**Tolerant**	**Tolerant**	Susceptible	Susceptible
1201K-1	4	3	Susceptible	**Resistant**	Susceptible	**Tolerant**	Susceptible
29275S-1	4	3	Susceptible	**Resistant**	Susceptible	Susceptible	**Tolerant**
365K-1	4	3	Susceptible	**Tolerant**	Susceptible	Susceptible	Susceptible
29275S-4	4	3	Susceptible	Susceptible	Susceptible	**Resistant**	Susceptible
376K-1	4	3	Susceptible	**Tolerant**	Susceptible	Susceptible	Susceptible
1438K-1	4	3	Susceptible	Susceptible	Susceptible	**Resistant**	Susceptible
1154K-1	4	3	Susceptible	**Resistant**	Susceptible	Susceptible	Susceptible
660K-1	4	3	Susceptible	**Tolerant**	Susceptible	Susceptible	Susceptible
401K-1	4	3	Susceptible	**Resistant**	Susceptible	**Resistant**	Susceptible
29364S-2	4	3	Susceptible	Susceptible	Susceptible	Susceptible	Susceptible
376K-7	4	3	Susceptible	Susceptible	Susceptible	**Resistant**	Susceptible
Calcutta 4	2	3	**Resistant**	**Resistant**	Susceptible	**Resistant**	**Resistant**
Kasaska	2	3	Susceptible	Susceptible	Susceptible	**Resistant**	Susceptible
Borneo	2	3	**Resistant**	Susceptible	Susceptible	Susceptible	Susceptible
716	2	4	Susceptible	**Tolerant**	Susceptible	Susceptible	Susceptible
2905	2	4	Susceptible	**Tolerant**	Susceptible	Susceptible	Susceptible
201087K-3	2	4	Susceptible	**Resistant**	Susceptible	**Resistant**	Susceptible
TMB2x 9172	2	4	Susceptible	**Resistant**	Susceptible	**Resistant**	Susceptible
1297−3	2	4	Susceptible	**Resistant**	Susceptible	**Resistant**	Susceptible
1448−1	2	4	Susceptible	**Resistant**	Susceptible	**Tolerant**	Susceptible
2829−62	2	4	Susceptible	Susceptible	Susceptible	**Tolerant**	Susceptible
TMB2x 6142	2	4	Susceptible	Susceptible	**Resistant**	**Tolerant**	**Tolerant**
201087K-4	2	4	Susceptible	Susceptible	Susceptible	Susceptible	Susceptible
TMB2x 8075−7	2	4	**Tolerant**	**Resistant**	Susceptible	Susceptible	Susceptible
IITA hybrid 2145/1320	2	4	Susceptible	Susceptible	Susceptible	Susceptible	Susceptible
25447-S7	2	4	Susceptible	Susceptible	Susceptible	**Tolerant**	Susceptible
5610S-1	2		Susceptible	**Resistant**	Susceptible	**Resistant**	Susceptible
SH 3217	2		Susceptible	Susceptible	Susceptible	**Resistant**	Susceptible

**Note:** The classifications above were reconstructed directly from the point estimates and 95% confidence intervals in forest plots.

Resistant and tolerant responses to the five biotic constraints were observed in multiple clusters (Fig 1; Table 2). For *R*. *similis*, resistant genotypes occurred in Clusters 1, 3 and 4, including 25974S-17, 1201K-1, 29275S-1, 201087K-3, TMB2x 9172 and 1297−3 (Table 2). For *Xvm*, resistance was recorded in cv. Rose (Cluster 2) and TMB2x 6142 (Cluster 4), while Zebrina GF (Cluster 2) and TMB2x 6142 (Cluster 4) were tolerant (Table 2). Resistance to *FOC*-R1 was also distributed across several clusters, including PITA 5 (Cluster 1), 201071K (Cluster 2), 29275S-4 and 1438K-1 (Cluster 3), and 201087K-3, TMB2x 9172 and 1297−3 (Cluster 4) (Table 2). For *P*. *fijiensis*, resistant and tolerant responses were observed in Clusters 2, 3 and 4 (Table 2). Tolerance to *C*. *sordidus* was observed in TMB2x 8075−7 (Cluster 4).

### Comparison of results from P-rep and RCBD designs

We compared weevil damage in genotypes common to the present study and previous studies that used an RCBD. The P-rep design produced substantially lower SEs (0.3–0.6) than those reported for RCBD (1.0–9.8) (Table 3), indicating greater precision in estimating genotype means. Genotype responses were broadly consistent across studies, with Mbwazirume classified as susceptible, Calcutta 4 and TMB2x 8075−7 as resistant, and SH 3142 as tolerant (Table 3). Differences observed for Obino Lewai and TMB2x 6142 may reflect variation in experimental conditions, response thresholds or phenotyping procedures among studies (Table 3). Overall, P-rep provided greater precision while maintaining the ability to discriminate among contrasting genotype responses.

**Table 3:** Comparison of banana weevil damage means, standard errors (SE), and response classifications for genotypes common to the present P-rep study and previous studies using RCBD.

Genotype	P-rep			RCBD			Literature
	Mean	SE	Response	Mean	SE	Response	
Mbwazirume	3.9	0.3	Susceptible	26.4	1.3	Susceptible	[10]
Obino Lewai	4.2	0.3	Susceptible	20.9	1.5	Intermediate	
TMB2x 6142	2.7	0.4	Tolerant	8.3	1.2	Resistant	
Calcutta 4	1.7	0.3	Resistant	8.4	1.1	Resistant	
TMB2x 8075−7	2.1	0.4	Resistant	7.3	1.0	Resistant	
TMB2x 7197−2	2.3	0.6	Resistant	7.3	1.2	Resistant	
Calcutta 4	1.7	0.3	Resistant	12.7	7.8	Resistant	[56]
SH 3142	3.1	0.3	Tolerant	52.5	9.8	Tolerant	

To illustrate the trade-off between genotype number and replication, we calculated the theoretical number of genotypes that could be accommodated using the same total number of plants in the P-rep for a balanced three-block design, assuming four plants per genotype per block. Under this hypothetical allocation, each genotype would require 12 plants, giving capacities of 75, 60, 22, 61 and 61 genotypes for *C. sordidus*, *R. similis*, *Xvm*, FOC-R1 and *P. fijiensis*, respectively (Table 4). This calculation highlights that the principal constraint addressed by the P-rep design was the limited and asynchronous availability of clonally propagated plants, rather than replication per se. A conventional RCBD requires each genotype to be represented in every replicate and, consequently, sufficient plants of each genotype to be available concurrently at the time of trial establishment. This requirement can substantially limit the number of genotypes that can be included when clonal multiplication rates differ among genotypes or plants become available at different times. In contrast, the P-rep design allowed genotypes to be incorporated as plants became available and allocated unevenly across blocks, enabling a more diverse set of genotypes to be screened while retaining replication for a subset of genotypes and all checks and controls. Thus, although the same total number of plants could theoretically accommodate a different number of genotypes under a conventional replicated design, the practical capacity of such a design is additionally constrained by the need for adequate numbers of plants per genotype to be available concurrently.

**Table 4:** Comparison of P-rep and conventional replication under an equivalent number of plants.

Constraint	P-rep		RCBD	
	No. of test plants	Genotypes evaluated	Plants/test genotype	Theoretical capacity
*C. sordidus*	899	65	12	75
*R. similis*	721	59	12	60
*Xvm*	267	64	12	22
*FOC*-R1	734	48	12	61
*P. fijiensis*	734	60	12	61

## Discussion

Resistant banana genotypes are a durable, cost effective option for resource constrained farmers and identifying them starts with controlled screenhouse phenotyping followed by multi- location field evaluations. This requires experimental designs with appropriate checks and controls, and statistical models that separate genetic effects from environmental noise. In screenhouse assays, responses are often influenced by microenvironment variation, inoculum quantification, and scoring methods making careful design essential for reliable inference. Effective control of microenvironments through experimental designs has long been emphasized [56–59].

Substantial variation was observed among the parental genotypes with some genotypes having resistance to more than one constraint. Such variation is valuable for breeding because resistant parental lines contribute resistance alleles to elite backgrounds while broadening the genetic base. However, reliable identification of such genotypes depends on phenotyping approaches that can distinguish genetic effects from environmental and experimental variation.

In this study, we adopted the P-rep design to capture genetic diversity that would be possible with full replication, while enabling unbiased estimation of genotype effects that might be confounded with other effects of the model. P-rep allows a smaller number of replications across many entries to achieve reliable and consistent results [60]. In banana, full replication is often unrealistic because of limited clonal material and associated costs of producing and maintaining sufficient plants [61]. Recent work continues to show that P-rep designs can be efficient and robust under unequal replication and, for resistance screening, can perform comparably to RCBD in detecting genotypic differences [25,29,62–66]. We phenotyped the parental genotypes in batches across five biotic constraints over a period of 36 months, and the density plots showed broadly consistent distributions among batches, supporting comparability of inference across screening cycles [25,62,64].

To allow for comparisons across batches, we selected documented, contrasting controls from previous evaluations; Calcutta 4 as the resistant control for most constraints [10,48,67–69], SH 3142 as an additional resistant reference for *R*. *similis* [70], and Monyet for partial resistance to *Xvm* [5]. Mbwazirume served as the routine susceptible check across multiple constraints of *C*. *sordidus*, *R*. *similis*, *Xvm* and *P*. *fijiensis* [71], with Obino Lewai as the classic plantain reference widely reported as susceptible to *P*. *fijiensis* and used as a female parent when making crosses for resistance breeding [72]. Mchare represented the local diploid cooking types and is susceptible to *FOC*-R1 and *P*. *fijiensis* [73]. Many of these genotypes have been phenotyped in the past and possibly do not need re-phenotyping. However, previous screening was possibly completed using different biotypes of the pathogens, under different conditions, experimental designs and analyses. We selected fewer checks and controls to avoid having more than two classes of phenotypic responses.

We adopted HGLM to evaluate contribution of fixed and random effects [73–76]. The forest plots clearly separated resistant parental genotypes from the susceptible ones, highlighting candidates for deployment as elite parents across the three breeding pipelines of Matooke, Mchare and Plantain. Overall, the findings highlight the need for carefully designed phenotyping protocols and robust experimental designs to reliably detect genotypic differences under biotic stress conditions [77,78]. In practice, consistent detection of genotypic variation is best achieved when disease pressure is uniform [79,80]. The controlled trials ensured uniformity of the disease and pest pressures. These findings highlight the importance of well thought out phenotyping protocols and experimental designs to accurately capture genotypic variation, when selecting resistant lines for use in breeding programs and highlight the essential role of using robust experimental designs in the development of resilient hybrids.

The resistance profiles provide a framework for selecting complementary parents for resistance pyramiding, prioritizing promising materials for pre-breeding, and designing targeted crosses according to the constraints of greatest breeding importance. Genotypes with resistance to several constraints can be prioritized as broad-spectrum donors, whereas materials with resistance to specific constraints can be used to complement existing parental resistance.

The integration of genetic clustering with resistance profiles revealed potentially valuable patterns for banana improvement. Genotypes exhibiting resistance or tolerance to the same biotic constraint within the same cluster may share genetic backgrounds and potentially resistance-associated alleles. Conversely, resistant or tolerant genotypes occurring in different clusters may represent genetically distinct sources of resistance, potentially involving different genes or allelic combinations. Although these patterns do not confirm distinct genetic mechanisms, they provide a basis for further genetic analysis and the selection of genetically diverse parents to broaden the genetic basis of resistance in banana.

The comparison with previous RCBD studies provides evidence that the P-rep design improves precision while using fewer plants per genotype. The substantially lower SEs obtained with P-rep indicate more precise estimation of genotype means than those reported from conventional RCBD experiments. Importantly, this gain in precision did not compromise the discrimination of genotypic responses, as several genotypes showed classifications consistent with previous studies. The comparison between P-rep and theoretical RCBD demonstrates the principal advantage of P-rep in accommodating unequal and asynchronous clone availability while retaining replication of reference genotypes. This is particularly relevant in banana breeding, where multiplication rates and plant availability can constrain simultaneous phenotyping of large number of genotypes. Thus, P-rep enables a greater number of genotypes to be evaluated with the available clonal material while maintaining replication of reference genotypes for biological interpretation. Overall, these findings support P-rep as an efficient design for phenotyping for resistance where the availability of clonally propagated planting material limits the use of fully replicated designs.

## Conclusion

This study demonstrated significant genotypic variation in banana resistance to *C. sordidus*, *R. similis*, *Xvm*, *FOC*-R1, and *P. fijiensis*, reinforcing the importance of robust phenotyping and experimental designs such as P-rep designs in resistance breeding. The P-rep design also provided greater precision in estimating genotypic responses, as reflected by the relatively low standard errors observed across the evaluated traits. The ability to detect consistent genotypic responses across independently phenotyped batches/cycles, together with improved precision, further supports the reliability and efficiency of the P-rep design for banana resistance phenotyping. The identification of genotypes with resistance to multiple constraints identified candidate genotypes for subsequent breeding. P-rep therefore offers a practical approach for phenotyping of banana breeding germplasm where uniform and fully replicated materials are difficult to obtain.

## Supporting information

S1 Table Banana genotypes commonly used as parents in the Matooke, Mchare and Plantain breeding pipelines. The land races that are used as susceptible checks are also included in the table. This table lists genotype names, their pedigree, genome groups and role either parent, landrace (Susceptible control) and resistant controls.
(DOCX)

S2 Table Batch-specific means and standard errors of control/check genotypes. This table presents the means and standard errors of response variables for the five biotic constraints across batches/cycles. %TRN, percentage total root necrosis; %TXD, percentage total cross-sectional damage; ICS, internal corm symptoms and AUDPC, Area under the disease progressive curve.
(DOCX)
