# Supplementary material for: Efficient phenotyping of banana (Musa spp.) germplasm for resistance to five biotic constraints using Partially replicated (P-rep) design
Source: PLoS One. 2026 Oct 23;21(10):e0361182. doi: 10.1371/journal.pone.0361182 (PMC13693891; doi:10.1371/journal.pone.0361182)
Supplement: Supplementary file 1 [file pone.0361182.s001.docx]

**Supplementary Table 1.** Banana genotypes commonly used as parents in the Matooke, Mchare and Plantain breeding pipelines. The landraces that are used as susceptible checks are also included in the table.

| **Genotype name** | **Female x Male** | **Genome group** | **Treatment/Control** |
| --- | --- | --- | --- |
| Calcutta 4 | Missing information | AA | Resistant |
| Mchare | Missing information | AA | Landrace |
| 201071K | 917K-2 x Unknown | AA | Parent |
| 2905 | Unknown | AA | Parent |
| CV. Rose | Missing information | AA | Parent |
| 1297-3 | French reversion x Calcutta 4 | AA | Parent |
| Zebrina GF | Missing information | AA | Parent |
| SH3217 | SH2905 x SH2766 | AA | Parent |
| 25447-S7 | 2829-62 x 9128-3 | AA | Parent |
| Galeo | Missing information | AA | Parent |
| 402 | Unknown | AA | Parent |
| 10969S-1 | 376K-7 x TMB2X5105-1 | AA | Parent |
| 5610S-1 | Kabucuragye x 7197-2 | AA | Parent |
| 2829-62 | Bobby Tannap x Calcutta 4 | AA | Parent |
| 201087K-3 | 917K-2 x Unknown | AA | Parent |
| TMB2X 6142 | Nyamwihogora x Long Tavoy | AA | Parent |
| TMB2X 5265-1 | Tjau lagada x Calcutta 4 | AA | Parent |
| Kokopo | Missing information | AA | Parent |
| SH3362 | SH3217 x SH3142 | AA | Parent |
| Borneo | Missing information | AA | Parent |
| SH3142 | SH1734 x Pisang Jari Buaya | AA | Parent/Resistant |
| TMB2X 9172 | Missing information | AA | Parent |
| 201087K-4 | 917K-3 x Unknown | AA | Parent |
| 716 | Unknown | AA | Parent |
| Banksii 897 | Missing information | AA | Parent |
| 1448-1 | Obino Lewai x Calcutta 4 | AA | Parent |
| TMB2X 7197-2 | SH3362 x Long Tavoy | AA | Parent |
| TMB2X 8075-7 | SH3362 x Calcutta 4 | AA | Parent |
| TMB2X 9128-3 | Tjau Lagada x Pisang lilin | AA | Parent |
| Pisang lilin | Missing information | AA | Parent |
| Kasaska | Missing information | AA | Parent |
| Guyod | Missing information | AA | Parent |
| IITA hybrid 2145/1320 | OP Zebrina GF?? | AA | Parent |
| Kwaro | Missing information | AA | Parent |
| Pahang | Missing information | AA | Parent |
| Paliama | Missing information | AA | Parent |
| Truncata | Missing information | AA | Parent |
| Maleb | Missing information | AA | Parent |
| Kunnan | Missing information | AB | Parent |
| 25291-S89 | 2829-62x 9128-3 | AA | Parent |
| Obino Lewai | Missing information | AAB | Plantain Landrace |
| Mbwazirume | Missing information | AAA | Matooke Landrace |
| 1411K-1 | Tereza x Calcutta 4 | AAAA | Parent |
| 365K-1 | Kabucuragye x Calcutta 4 | AAAA | Parent |
| 1747K-1 | Entukura x Calcutta 4 | AAAA | Parent |
| PITA 11 (2637-49) | Obino Lewai x Calcutta 4 | AAAB | Parent |
| 660K-1 | Enzirabahima x Calcutta 4 | AAAA | Parent |
| 917K-2 | Enzirabahima x Calcutta 4 | AAAA | Parent |
| 917K-1 | Enzirabahima x Calcutta 4 | AAAA | Parent |
| PITA 8 (7002-1) | Obino Lewai x Calcutta 4 | AAAB | Parent |
| 222K-1 | Nfuuka x Calcutta 4 | AAAA | Parent |
| 29275S-1 | Enzirabahima x M. a. malaccensis 250 | AAAA | Parent |
| 376K-1 | Nante x Calcutta 4 | AAAA | Parent |
| PITA 4 (582-4) | Bobby Tannap x Calcutta 4 | AAAB | Parent |
| 1201K-1 | Nakawere x Calcutta 4 | AAAA | Parent |
| 29275S-4 | Enzirabahima x M. a. malaccensis 250 | AAAA | Parent |
| 376K-7 | Nante x Calcutta 4 | AAAA | Parent |
| PITA 6 (4698-1) | Obino Lewai x Calcutta 4 | AAAB | Parent |
| 246K-1 | Kabucuragye x Calcutta 4 | AAAA | Parent |
| 29364S-2 | Namwezi x CV. Rose | AAAA | Parent |
| PITA 7 (1658-4) | Obino Lewai x Pisang lilin | AAAB | Parent |
| PITA 5 (2796-5) | Bobby Tennap x Pisang lilin | AAAB | Parent |
| 1154K-1 | Nakayonga x Calcutta 4 | AAAA | Parent |
| 25974S-17 | 917K x SH3362 | AAAA | Parent |
| 1438K-1 | Entukura x Calcutta 4 | AAAA | Parent |
| 401K-1 | Entukura x Calcutta 4 | AAAA | Parent |
| PITA 1 (548-4) | Obino Lewai x Calcutta 4 | AAAB | Parent |
| PITA 2 (548-9) | Obino Lewai x Calcutta 5 | AAAB | Parent |
| PITA 12 (6930-1) | Obino Lewai x Calcutta 6 | AAAB | Parent |
| 29275S-5 | Enzirabahima x M. a. malaccensis 250 | AAAA | Parent |
| PITA 3 (5511-2) | Obino lewai x Calcutta 4 | AAAB | Parent |
| PITA 18 (7356-1) | Obino lewai x Calcutta 4 | AAAB | Parent |
| Monyet | Missing information | AAAA | Parent / Tolerant |
