# Supplementary material for: Efficient phenotyping of banana (Musa spp.) germplasm for resistance to five biotic constraints using Partially replicated (P-rep) design
Source: PLoS One. 2026 Oct 23;21(10):e0361182. doi: 10.1371/journal.pone.0361182 (PMC13693891; doi:10.1371/journal.pone.0361182)
Supplement: Supplementary file 2 [file pone.0361182.s002.docx]

**Supplementary Table 2:** Batch-specific means and standard errors of control/check genotypes

| **Biotic constraint** | **Batch/Cycle** | **Resistant checks** | | **Landraces / Susceptible controls** | | | |
| --- | --- | --- | --- | --- | --- | --- | --- |
|  |  | **Calcutta 4** | **Monyet** | **Mbwazirume** | **Obino Lewai** | **Mchare** | **Sukali ndiizi** |
| ***Radopholus similis*** (%TRN) | 1 | 10.71 ± 3.08 |  | 27.58 ± 3.75 | 27.92 ± 5.40 | 28.33 ± 4.13 |  |
|  | 2 | 5.27 ± 1.50 |  | 38.10 ± 7.39 | 10.13 ± 2.17 | 31.17 ± 3.97 |  |
|  | 3 | 2.75 ± 1.45 |  | 31.69 ± 7.25 | 12.94 ± 2.71 | 11.15 ± 1.71 |  |
|  | 4 | 5.00 ± 2.73 |  | 8.50 ± 6.17 | 16.25 ± 2.47 | 18.92 ± 6.75 |  |
| ***Cosmopolites sordidus*** (%TXD) | 1 | 3.28 ± 1.14 |  | 61.19 ± 6.65 | 73.89 ± 7.66 | 32.20 ± 4.49 |  |
|  | 2 | 5.67 ± 1.59 |  | 51.90 ± 8.86 | 91.03 ± 4.38 | 31.56 ± 6.78 |  |
|  | 3 | 8.92 ± 3.01 |  | 39.79 ± 7.73 | 31.44 ± 6.78 | 37.25 ± 7.88 |  |
|  | 4 | 5.75 ± 3.25 |  | 66.43 ± 5.24 | 48.50 ± 21.22 | 55.27 ± 5.72 |  |
|  | 5 | 2.06 ± 0.72 |  | 67.44 ± 6.60 | 88.13 ± 4.73 | 52.22 ± 7.68 |  |
| ***Xanthomonas vasicola* pv. *musacearum*** (AUDPC) | 1 |  | 243.06 ± 9.72 | 281.94 ± 35.05 | 350.00 ± 0.00 | 350.00 ± 0.00 |  |
|  | 2 |  | 204.17 ± 16.84 | 301.39 ± 35.05 | 301.39 ± 25.72 | 350.00 ± 0.00 |  |
|  | 3 |  | 233.33 ± 16.84 | 239.81 ± 81.02 | 291.67 ± 58.33 | 350.00 ± 0.00 |  |
|  | 4 |  | 158.80 ± 40.61 | 291.67 ± 58.33 | 233.33 ± 0.00 | 350.00 ± 0.00 |  |
|  | 5 |  | 213.89 ± 35.05 | 272.22 ± 9.72 | 330.56 ± 9.72 | 330.56 ± 19.44 |  |
| ***Fusarium oxysporum* f. sp. *cubense* Race 1** (ICS) | 1 | 1.00 ± 0.00 |  |  |  | 6.00 ± 0.00 | 5.50 ± 0.22 |
|  | 2 | 1.00 ± 0.00 |  |  |  | 6.00 ± 0.00 | 4.33 ± 1.05 |
| ***Pseudocercospora* *fijiensis*** (AUDPC) | 1 | 52.88 ± 16.31 |  | 86.10 ± 16.84 | 113.14 ± 25.92 | 188.69 ± 24.49 |  |
|  | 2 | 67.44 ± 16.52 |  | 103.67 ± 10.52 | 107.05 ± 23.84 | 112.54 ± 28.94 |  |
|  | 3 | 66.05 ± 16.22 |  | 105.76 ± 14.83 | 147.08 ± 24.70 | 168.81 ± 24.85 |  |
